# Structure Activity Relationship of *N*-Substituted Phenyldihydropyrazolones Against *Trypanosoma cruzi* Amastigotes

**DOI:** 10.3389/fchem.2021.608438

**Published:** 2021-04-30

**Authors:** Maarten Sijm, Louis Maes, Iwan J. P. de Esch, Guy Caljon, Geert Jan Sterk, Rob Leurs

**Affiliations:** ^1^ Division of Medicinal Chemistry, Faculty of Sciences, The Amsterdam Institute of Molecular and Life Sciences (AIMMS), Vrije Universiteit Amsterdam, Amsterdam, Netherlands; ^2^ Laboratory for Microbiology, Parasitology and Hygiene (LMPH), University of Antwerp, Antwerp, Belgium

**Keywords:** structure activity relationship, chagas disease, phenotypic optmization, trypanosama cruzi, phenylpyrazolones

## Abstract

Current drugs for Chagas disease have long treatment regimens with occurrence of adverse drug effects leading to poor treatment compliance. Novel and efficacious medications are therefore highly needed. We previously reported on the discovery of NPD-0227 (2-isopropyl-5-(4-methoxy-3-(pyridin-3-yl)phenyl)-4,4-dimethyl-2,4-dihydro-3H-pyrazol-3-one) as a potent *in vitro* inhibitor of *Trypanosoma cruzi* (pIC_50_ = 6.4) with 100-fold selectivity over human MRC-5 cells. The present work describes a SAR study on the exploration of substituents on the phenylpyrazolone nitrogen. Modifications were either done directly onto this pyrazolone nitrogen or alternatively by introducing a piperidine linker. Attention was pointed toward the selection of substituents with a cLogP preferably below NPD-0227s cLogP of 3.5. Generally the more apolar compounds showed better activities then molecules with cLogPs <2.0. Several new compounds were identified with potencies that are in the same range as NPD-0227 (pIC_50_ = 6.4) and promising selectivities. While the potency could not be improved, valuable SAR was obtained. Furthermore the introduction of a piperidine linker offers new opportunities for derivatization as valuable novel starting points for future *T. cruzi* drug discovery.

## Introduction

The protozoan parasite *Trypanosoma cruzi* is the causative agent of Chagas disease. It is estimated that over six million people are infected worldwide, the majority in Latin-America where the parasite is endemic. (Lidani et al., 2019; WHO, 2021). *T. cruzi* is spread by several insect vectors, most of which belong to the triatomine family (Kollien and Schaub, 2000; Martinez et al., 2019). Other ways of transmission are *via* blood transfusions, laboratory accidents or parental transmission from mother to infant (Schmunis, 1999; Torrico et al., 2004; Rassi et al., 2010). The life cycle of *T. cruzi* consists of several developmental stages in the mammalian host and insect vector (Rassi et al., 2010). While the vector appears unaffected by the parasite, infected humans can develop life-threatening symptoms and pathologies. The acute stage of the disease starts shortly after the parasite enters the body but a strong immune response will cause a large reduction of the initial peak of parasitemia, however, without full elimination of the parasite leading to persistent infection of certain tissues (Dias, 1984). The acute symptoms are non-specific, such as fever, mild splenomegaly and edema, making diagnosis diffucult (Prata, 2001). After 2 months, the disease enters the chronic phase in which the parasite becomes dormant and no symptoms are observed. This dormant phase can last for over 10 years up to lifelong (Prata, 2001). Estimates of further evolution of chronic infection toward clinical symptoms, such as cardiopathy or megaesophagus and megacolon, ranges between 10–60% of infected people and varies widely in different regions (Coura and Viñas, 2010; Coura and Borges-Pereira, 2011). Patients can become life-long asymptomatic carriers thus representing a parasite reservoir.

**FIGURE 1:**
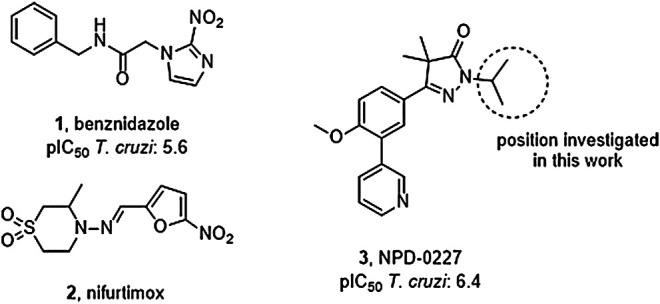
Current drugs used against Chagas disease: the nitro-imidazole benznidazole (**1**) and nitro-furan nifurtimox (**2**), and the recently discovered *T. cruzi* inhibitor NPD-0227 (**3**).

Vector control, early diagnosis and effective chemotherapy are essential to combat Chagas disease. Two drugs are currently marketed: the nitro-imidazole benznidazole (**1,** Figure 1) and the nitro-furan nifurtimox (**2**) (Rassi et al., 2010). Both drugs contain a nitro-aromatic functionality which is commonly associated with toxic side effects. Furthermore, the treatment regimens of both vary between 60–90 days and are known to cause various side effects which cause patients to discontinue their treatment (Castro et al., 2006; Pinazo et al., 2013). Benznidazole (**1**) is currently used as first-line treatment as it has a better overall safety profile (Alpern et al., 2017). While the efficacy of both drugs is well established in the acute phase, much debate is ongoing on about their efficacy in the chronic phase (Sgambatti de Andrade et al., 1996; Zhang and Tarleton, 1999; Bern, 2011). The recent BENEFIT trial investigated the efficacy of Benznidazole (**1**) on chronic Chagas’ heart disease. While reduced parasitemia was observed by PCR, this did not result in significant reduction of clinical deterioration of cardiac function after 5 years (Morillo et al., 2015). Afterward several concerns were raised in literature with regard to the PCR analysis and benznidazole dosage, precluding firm conclusions on the efficacy potential in the chronic phase of infection (Hamers et al., 2016).

The overall need for new and improved chemotherapies for Chagas disease is high, both for current and future patients. New medication is also needed as contingency measure for upcoming resistance against the available current drugs (Coura and Viñas, 2010; Mejia et al., 2012). Unfortunately, the pipeline toward new drugs is largely empty. Most research on *T. cruzi* is performed in academia though the last few years several public-private collaborations have been initiated. In this paper, we describe part of the research of the EU-funded, public-private consortium PDE4NPD, that focuses on 3′5′-cyclic nucleotide phosphodiesterases (PDEs) as targets against several neglected tropical diseases. The present work further elaborates the SAR of the previously reported *T. cruzi* inhibitor NPD-0227 (**3**, Figure 1) and investigates the role of different substituents on the pyrazolone nitrogen (Sijm et al., 2019). Besides the SAR aiming at improving potency, compounds were also specifically designed to improve physicochemical properties, such as cLogP and solubility. As *T. cruzi* ultimately proceeds *via* a dormant intracellular form, a possible drug needs to pass several cell membranes before reaching the parasite. During this process the drug is transferred multiple times between hydrophobic and hydrophilic phases, as such an optimal cLogP is thought to be between 0 and 2 and the drug having no charges (Basore et al., 2015; Bennion et al., 2017).

### Chemistry

**SCHEME 1:**
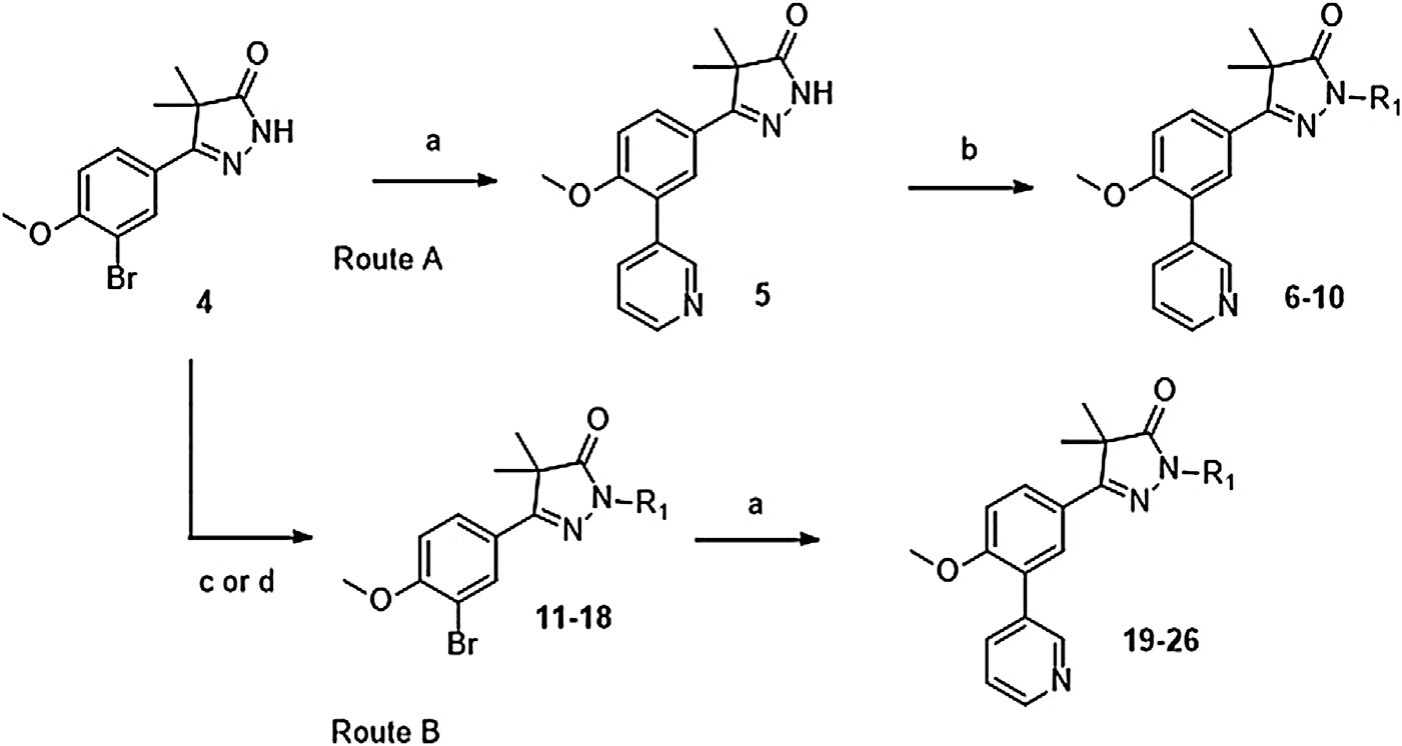
Reagents and conditions: a: 3-pyridinyl-B (OH)_2_, Pd (dppf)Cl_2_. DCM, Na_2_CO_3_, DME:H_2_O, 120 °C, 1 h, 12–78%, b: R-COCl (**6**, **7**, **8**) or R-SO_2_Cl (**9**, **10**), NaH, DMF, rt, 18 h, 39–77%, c: epoxide (**11**–**12**), K_2_CO_3_, DMF, 100 °C, 16 h, 59–63%, d: R-Br (**13**–**17**) or R-Cl (**18**), NaH, DMF, rt, 18 h, 36–94%.

Phenylpyrazolone analogues with a substituent directly on the pyrazolone nitrogen atom were synthesized using two different routes. The first route started with a Suzuki reaction to obtain dihydropyrazolone **5** (Route A, Scheme 1) which was then functionalized using sodium hydride and various acyl chlorides and sulfonylchlorides to obtain compounds **6**–**10** (Table 1). The second route (Route B) started with alkylation of the dihydropyrazolone **4**, either using potassium carbonate to yield intermediates **11**–**12**, or using sodium hydride to generate intermediates **13**–**18**. These intermediates were then converted to the 3-pyridinyl derivatives *via* a Suzuki cross coupling, yielding final compounds **20**–**26** (Scheme 1, Table 1).

**TABLE 1:** *In vitro* activity against intracellular amastigotes of *T.cruzi* (Tulahuen strain)^a^ and MRC-5 cells^a^ of phenyldihydropyrazolones with modifications directly on the pyrazolone nitrogen.

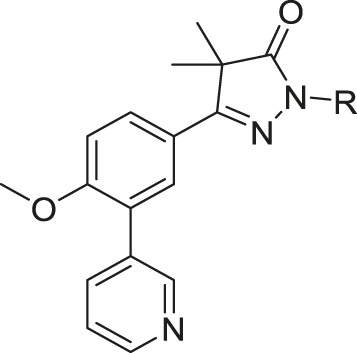											

Cpd	R	*T.cruzi* (pIC_50_)	MRC-5 (pIC_50_)	SI^b^	cLogP	Cpd	R	*T.cruzi* (pIC_50_)	MRC-5 (pIC_50_)	SI^b^	cLogP
3	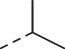	6.4	4.4	100	3.5	22	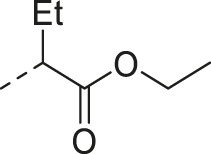	5.2	<4.2	>10	3.4
6	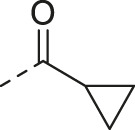	4.4	<4.2	>2	3.2	23	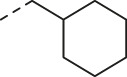	5.6	4.9	5	4.8
7	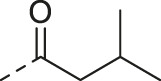	4.5	<4.2	>2	3.9	24	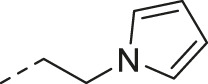	5.5	4.5	10	3.8
8	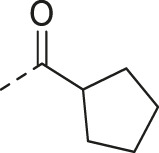	4.4	<4.2	>2	4.1	25	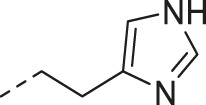	4.5	<4.2	>2	2.3
9	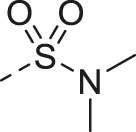	4.4	<4.2	>2	1.9	26	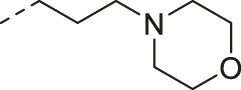	<4.2	<4.2	N.D.	2.5
10	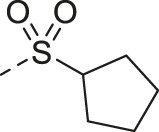	5.0	4.4	4	3.6	33	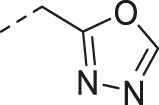	5.1	<4.2	>8	1.5
19	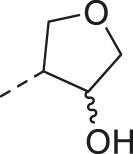	4.4	<4.2	>2	1.9	34	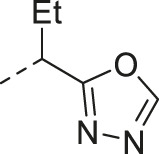	5.2	<4.2	>10	2.5
20	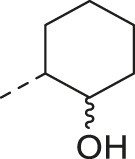	5.1	<4.2	>8	3.4	35	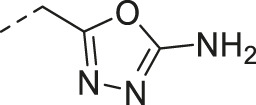	4.2	4.2	0	1.3
21	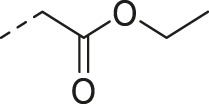	5.2	<4.2	10	2.6	36	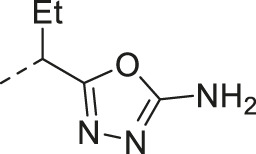	4.4	<4.2	2	2.4

a All reported values are measured as duplicates and had a standard deviation less then ±0.2.

b Selectivity index was calculated as IC_50_ MRC-5 cells divided by IC_50_ *T. cruzi*.

**SCHEME 2:**
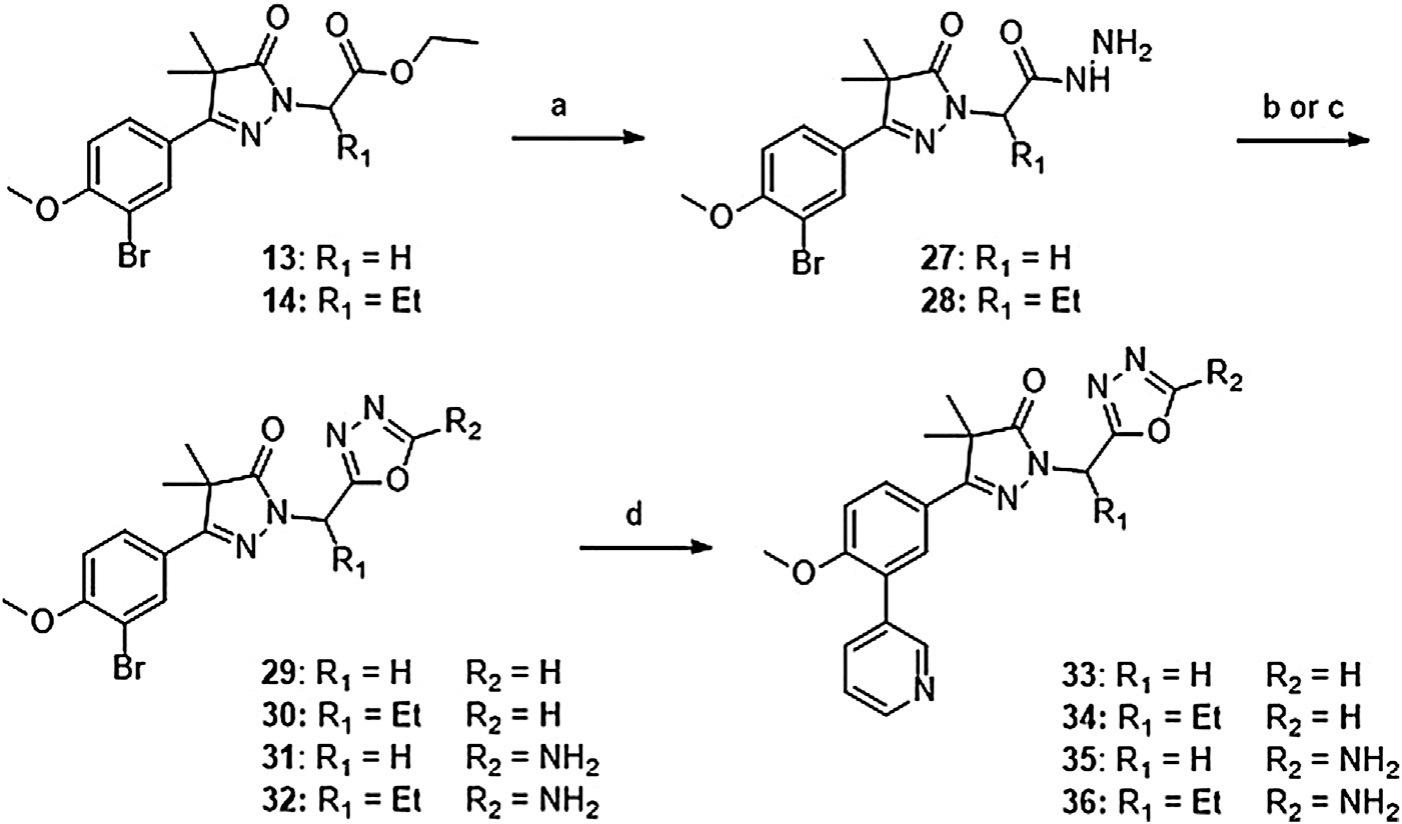
Reagents and conditions: a: N_2_H_4_, EtOH, rt, 18 h, 90–94%, b: (EtO)_3_CH, reflux,18 h, 34–78% (**29**–**30**), c: BrCN, NaHCO_3_, MeOH, H_2_O, o. n., 22–46% (**31**–**32**), d: 3-pyridinyl-B (OH)_2_, Pd (dppf) Cl_2_. DCM, Na_2_CO_3_, DME:H_2_O, 120 °C, 1 h 36–74%.

Introduction of methylene-oxadiazoles on the pyrazolone head group started with the previously synthesized ester analogues **13** and **14** (Scheme 2). Subsequent hydrazination yielded the corresponding hydrazides (**27** and **28**) which were ring-closed with either triethylorthoformate or cyanuric bromide to deliver the unsubstituted oxadiazoles (**29** and **31**) and the amine substituted oxadiazoles (**30** and **32**) respectively. These oxadiazoles were then converted to the corresponding 3-pyridinyl analogues *via* a Suzuki cross-coupling to yield compounds **33**–**36**.

**SCHEME 3:**
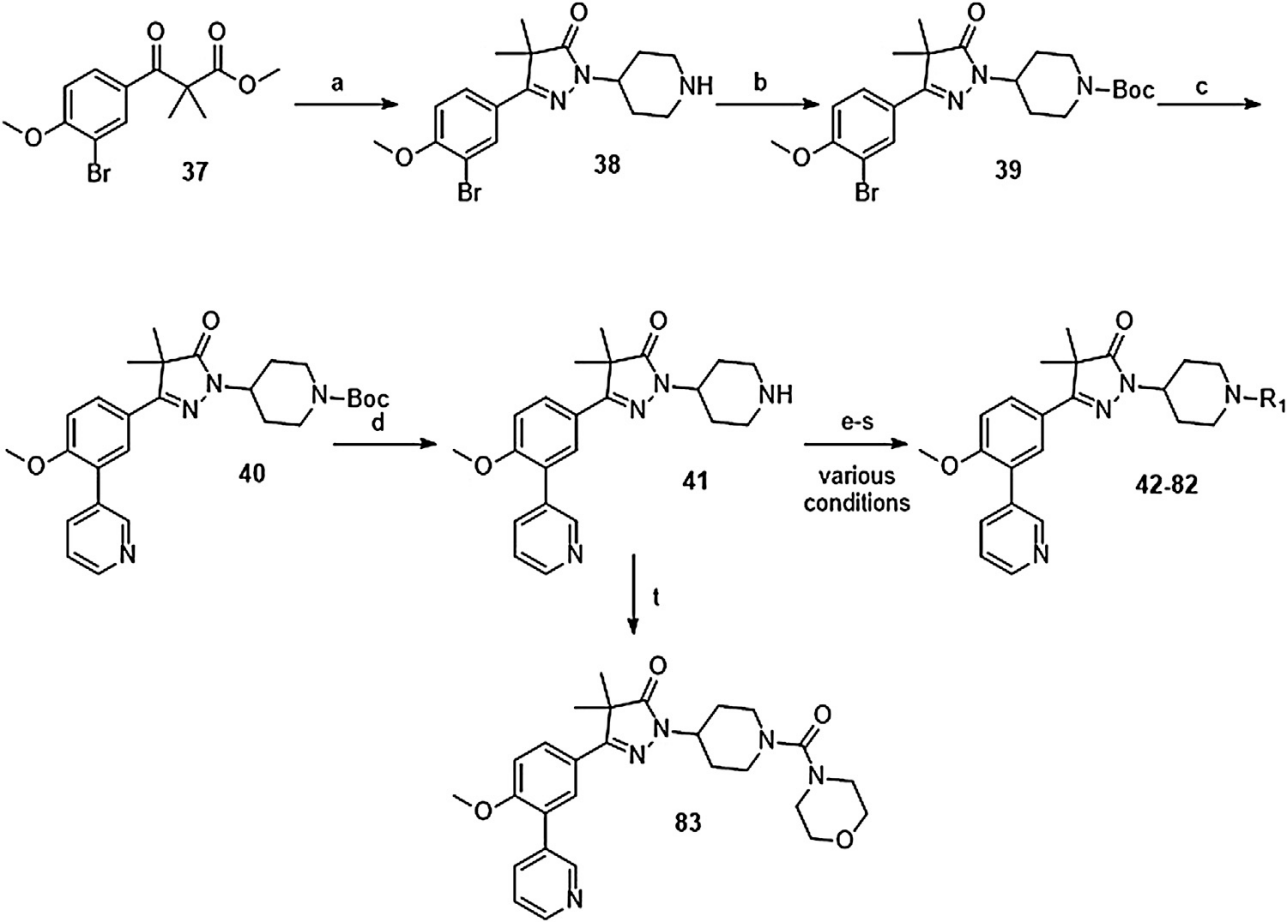
Reagents and conditions: a: 4-hydrazinylpiperidine.2HCl, MeOH, H_2_O, reflux, 3 days, 80%, b: Boc_2_O, TEA, DCM, rt, 4 h, 89%, c: 3-pyridinyl-B (OH)_2_, Pd (dppf) Cl_2_. DCM, Na_2_CO_3_, DME:H_2_O, 120 °C, 1 h, 86%, d: 4 M HCl in dioxane, rt, 18 h, 84%, e: R-COCl (**42**, **43**), R-SO_2_Cl (**48**), NaH, DMF, rt, 2 h, 38–42%, f: EDCI, HOBt, RCOOH (**44**, **46**), DIPEA, DCM, 18–30 h, 23–31%, g: oxazole-5-carboxylic acid (**45**), T3P, DIPEA, EtOAc, 50 °C, 70 h, 7%, h: R-COCl (**47**) or R-SO_2_Cl (**49**–**54**) or OCN-R (**55**, **57**–**59**, **61**) or Cl (CO)NR_1_R_2_ (**56**, **60**), TEA, DCM, rt, 2–42 h, 13–79%, i: epoxide (**62**–**66**), DMAP, i-PrOH, 50–100 °C, 2–120 h, 4–30%, j: formic acid (**67**), formaldehyde, 18 h, rt, 15%, k: R-CO-R (**68–69**, **71**, **74**), NaBH (OAc)_3_, AcOH, DCE, 70 °C, 18–72 h, 10–53%, l: R-Br (**70**), K_2_CO_3_, DMF, rt, 48 h, 10%, m: methylacrylate (**72**), DBN, ACN, 90h, rt, 45%, n: ClCH_2_CO-R (**73**), K_2_CO_3_, ACN, 24 h, rt, 39%, m: HCO-R (**74**–**78**), NaBH (OAc)_3_, AcOH, DCE, 22–72 h, 5–48%, q: Ar-Br (**79**–**80**), Pd_2_ (dba)_3_, BINAP, NaOtBu, 80 °C, 7 days, 11–19%, r: Cl-Ar (**81**), Cs_2_CO_3_, DMF, 90 °C, 4 h, 24%, s: F-Ar (**82**), DMSO, K_2_CO_3_, 110 °C, 6 days, 16% t: i: (**83**) NaHCO_3_, 4-NO_2_Ph-chloroformate, dioxane, rt, 22 h, ii: K_2_CO_3_, morpholine, DMF, rt, 4 days.

The synthesis of the piperidine substituted dihydropyrazolones started with keto-ester **37** (Scheme 3) which was prepared according to previously reported methodology (Sijm et al., 2019). This keto-ester was condensed with 4-hydrazinylpiperidine to yield piperidine substituted dihydropyrazolone **38**. The piperidine nitrogen atom was protected using boc-anhydride to give intermediate (**39**) which could be used in a Suzuki cross-coupling to install the 3-pyridine moiety. Subsequent deprotection of **40** with 4 M HCl in dioxane yielded the free amine building block (**41**) which was used to install the desired electrophiles (**42**–**83**, Tables 2,3) *via* various methodologies.

**TABLE 2:** *In vitro* activity against intracellular amastigotes of *T.cruzi* (Tulahuen strain)^a^ and MRC-5 cells^a^ of phenyldihydropyrazolones with a piperidine linker.

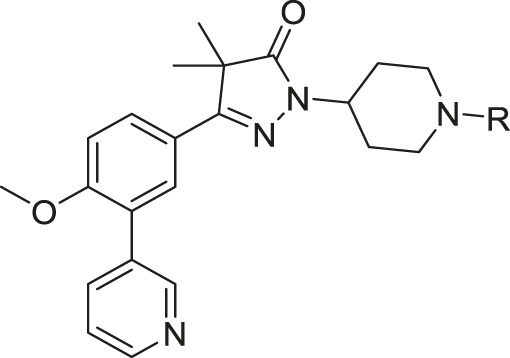											

Cpd	R	*T.cruzi* (pIC_50_)	MRC-5 (pIC_50_)	SI^b^	cLogP	Cpd	R	*T.cruzi* (pIC_50_)	MRC-5 (pIC_50_)	SI^b^	cLogP
40	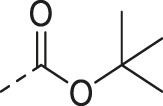	5.9	<4.2	>50	3.6	52	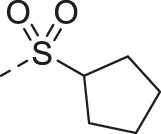	5.2	4.5	5	3.1
42	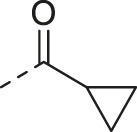	4.4	<4.2	>2	2.7	53	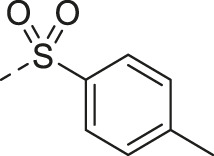	<4.2	<4.2	N.D.	4.4
43	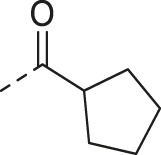	5.2	4.5	5	3.6	54	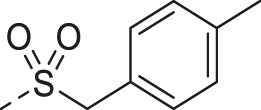	6.2	<4.2	>100	3.8
44	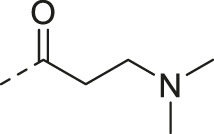	4.5	<4.2	>2	2.1	55	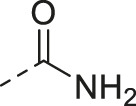	<4.2	<4.2	N.D.	1.6
45	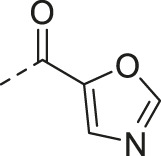	4.6	<4.2	>3	1.6	56	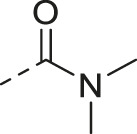	4.5	<4.2	>2	2.0
46	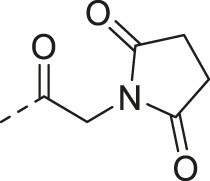	<4.2	<4.2	N.D.	0.9	57	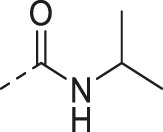	4.9	<4.2	>5	2.6
47	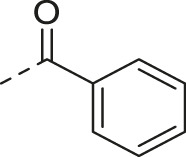	<4.2	<4.2	N.D.	3.8	58	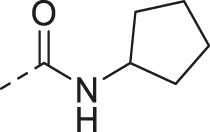	5.5	4.5	10	3.2
48	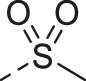	4.5	<4.2	>2	1.4	59	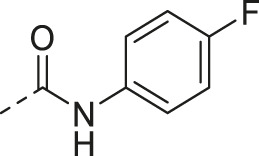	5.9	4.5	25	4.0
49	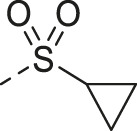	5.0	<4.2	>6	2.2	60	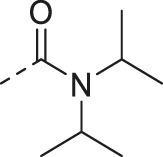	<4.2	<4.2	N.D.	3.6
50	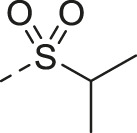	5.1	<4.2	>8	2.5	61	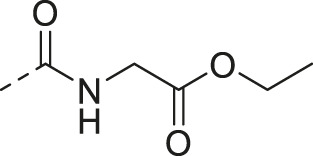	<4.2	<4.2	N.D.	1.8
51	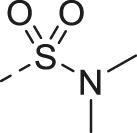	5.1	<4.2	>8	1.4	83	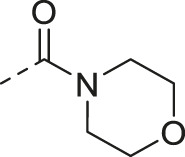	4.9	<4.2	>5	1.8

a All reported values are measured as duplicates and had a standard deviation less then ±0.2.

b Selectivity index was calculated as IC_50_ MRC-5 cells divided by IC_50_ *T. cruzi*.

**TABLE 3:** *In vitro* activity against intracellular amastigotes of *T.cruzi* (Tulahuen strain)^a^ and MRC-5 cells^a^ toxicity by phenyldihydropyrazolones with a piperidine linker.

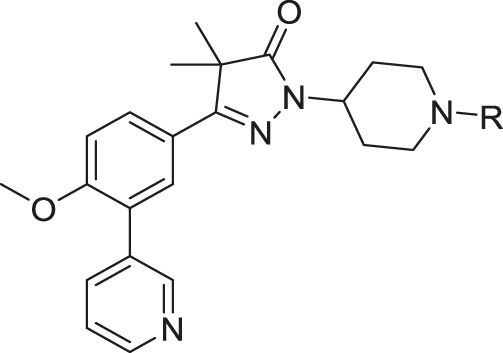											

Cpd	R	*T.cruzi* (pIC_50_)	MRC-5 (pIC_50_)	SI^b^	cLogP	Cpd	R	*T.cruzi* (pIC_50_)	MRC-5 (pIC_50_)	SI^b^	cLogP
41	H	<4.2	<4.2	N.D	2.3	72	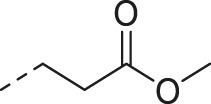	<4.2	<4.2	N.D.	2.6
62	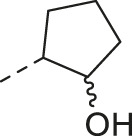	4.5	4.5	1	3.0	73	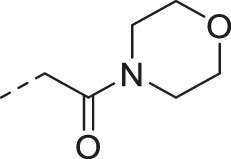	4.4	<4.2	>2	1.6
63	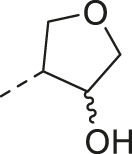	<4.2	<4.2	N.D	1.9	74	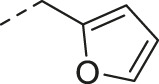	5.0	4.6	3	3.5
64	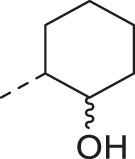	4.5	4.6	<1	3.4	75	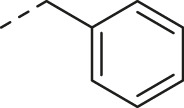	5.6	4.6	10	4.4
65	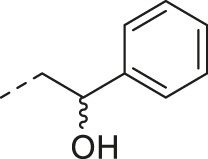	5.6	<4.2	>25	3.8	76	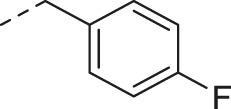	6.1	5.1	10	4.6
66	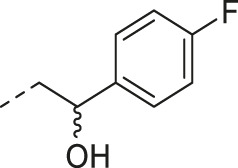	5.7	5.2	3	3.9	77	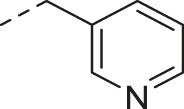	5.3	4.5	6	3.2
67	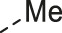	4.4	<4.2	>2	2.7	78	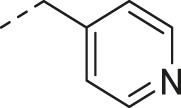	5.7	4.5	16	3.2
68	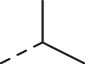	4.5	<4.2	>2	3.5	79	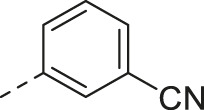	5.7	<4.2	>32	4.4
69	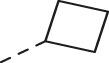	4.9	4.5	3	3.6	80	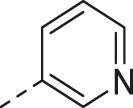	5.2	4.5	5	3.4
70	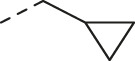	4.8	4.5	2	3.5	81	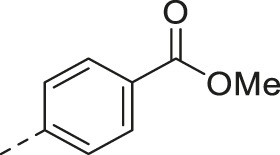	5.7	5.0	5	4.6
71	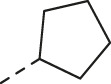	4.6	4.5	1	4.1	82	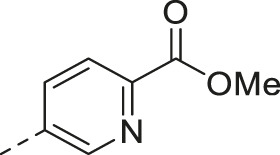	5.7	<4.2	>32	4.0

a All reported values are measured as duplicates and had a standard deviation less then ±0.2.

b Selectivity index was calculated as IC_50_ MRC-5 cells divided by IC_50_ *T. cruzi*.

### Pharmacology and Parasitology

All compounds were tested for their trypanocidal activity against intracellular forms of *T. cruzi* (Tulahuen CL2, β-galactosidase strain (drug sensitive strain (discrete typing units, DTU VI)) as well as for cytotoxicity on MRC-5_SV2_ cells (human lung fibroblasts). (Blaazer et al., 2014).

## Results and Discussion

In the previous work by Sijm *et al.*, the pyrazolone nitrogen was substituted with various (cyclo)-alkyl moieties resulting in the identification of NPD-0227 (**3**) as a potent (pIC_50_ = 6.4) *T. cruzi* inhibitor with 100-fold selectivity over human MRC-5 cells (Table 1) (Sijm et al., 2019; Sijm et al., 2020). To further explore SAR and to investigate molecules with varying cLogPs, more polar and diverse substituents was explored.

Introduction of a carbonyl-alkyl moieties directly onto the pyrazolone nitrogen (**6–8**, Table 1) resulted in weak inhibitors with pIC_50_’s around 4.5. Similarly, introduction of sulphonyl-alkyl moieties such as dimethylsulfonamide (**9**) or sulphonylcyclopentyl (**10**) did not lead to inhibitors with pIC_50_ values above 5.0. Epoxides were used to introduce β-alcohols on the pyrazolone ring resulting in compounds **19** and **20**. These alcohols also showed a large decrease in activity compared to NPD-0227 (pIC_50_ = 6.4) with pIC_50_’s of 4.4 and 5.1, respectively. Similarly, the introduction of ethyl-esters (**21**, **22**) resulted in decreased potencies compared to NPD-0227 with both compounds showing pIC_50_ values of only 5.2.

While the substituents with a polar moiety (**6**–**10**, **19**–**22**, Table 1) showed decreased activities, as expected, introduction of more apolar moieties such as methyl-cyclohexyl (**23**) and pyrazole (**24**) showed moderate activity with pIC_50_ values of 5.5. Introduction of more polar substituents attached to an aliphatic linker, such as imidazole **25** and morpholine **26** with cLogPs around 2.3 resulted in lower potencies (pIC_50_ < 4.6). While the introduction of various oxadiazoles (**33**–**36**) on this position yielded compounds with a more desirable cLogP’s, this did not lead to very active compounds, with 1-propan-oxazole (**34**) showed the highest potency (pIC_50_ = 5.2). As activities were generally close to the lower detection limit of the assay, selectivity indexes were quite low or defined as “bigger then”.

The initial attempts to introduce polar functional groups such as carbonyls, sulphonyls and alcohols close to the pyrazolone ring had a negative effect on their potency. One of the most potent compounds so far was methylcyclohexyl **23**, a bulky and apolar substituent. It was thought that introduction of a piperidine would introduce a similar sized moiety which would improve options to introduce polarity as a new handle to further modify.

This piperidine linker was first investigated by forming amides with corresponding acid chlorides or carboxylic acids, which led to analogues **42**–**47** (Table 2). These groups introduced some polarity but these modifications did not lead to potent ligands (pIC50 < 5.3). Introducing sulphonamides instead of regular amides gave similar results as the majority showed moderately low activity (**48**–**54**). Alkyl-sulphonamides with a cyclopropyl (**49**), isopropyl (**50**), dimethylamino (**51**) and cyclopentyl (**52**) all showed pIC_50_’s around 5.1, while the smaller methyl substituted sulphonamide (**48**) had a slightly lower activity (pIC_50_ = 4.5). Introduction of an aromatic ring on to the sulphonamide did not have a positive effect. Tosylate **53** (cLogP = 4.4) was inactive with a pIC_50_ below 4.2. Introduction of an extra carbon between the sulphonamide and the benzene ring was successful as methylbenzyl **54** (cLogP = 3.8) showed a similar potency as NPD-0227 (**3**, Table 1) with a pIC_50_ value of 6.2 and >100-fold selectivity over human MRC-5.

The boc-protected analogue **40** (cLogP = 3.6; pIC_50_ = 5.9, Table 2) which was obtained during the synthesis of the free-amine building block showed that relatively large substituents are allowed on the piperidine linker, which was confirmed by sulphonamide **54**. The preference for larger, bulky substituents is also apparent with the urea linked analogues (**55–59**). While the carbamide is inactive (**55**, cLogP = 1.6; pIC_50_ = <4.2), introduction of larger apolar groups increased activity (**57–59**), with the most potent compound being the 4-fluorophenyl **59** (pIC_50_ = 5.9; cLogP = 4.0, 25-fold selectivity). The even more bulky disopropyl analogue (**60**), however, was totally inactive (pIC_50_ < 4.2). Introduction of more polar substituents attached to the urea linker did not improve activity with pIC_50_ values of <4.2 for ethylacetate derivative **61** (cLogP = 1.8) and 4.9 for the morpholine analogue (**83**, cLogP = 1.8). Two types of epoxides were used to introduce β-alcohols. While the aliphatic analogues **62**–**64** (Table 3) showed low potencies (pIC_50_ < 4.6), the aromatic derivatives (**65**–**66**) showed micromolar activities with pIC_50_ values of 5.6 and 5.7. Selectivity of the unsubstituted phenyl ring (**65**) was however a lot better than the 4-fluorophenyl (**66**).

As shown in Table 1 and in previous work, aliphatic substituents on the dihydropyrazolone nitrogen led to the most potent compounds (Sijm et al., 2019). Introduction of aliphatic substituents on the piperidine nitrogen resulted in poor potencies, possibly caused by the introduction of a tertiary amine which can be protonated, as charged compounds are less likely to cross the cell membrane. The unsubstituted piperidine (**41**, cLogP = 2.3, Table 3) is totally inactive (pIC_50_ < 4.2) and the alkylated analogues (**67**–**73**) are not very active inhibitors (pIC_50_ < 5.0). Analogues with an aromatic ring showed higher activities: the 2-furanylmethyl (**74**, cLogP = 3.5) and the 3-pyridineylmethyl (**77**, cLogP = 4.4) showed pIC_50_ values of 5.0 and 5.3, respectively. The benzyl substituted piperidine **75** and the 4-pyridinemethyl **78** both showed pIC_50_ values around 5.6, while 4-fluorobenzyl **76** (cLogP = 4.6) showed sub-micromolar potency with a pIC_50_ of 6.1. Selectivity indexes of these compounds were moderate to poor, with the best compound, 4-pyridinemethyl **78** showing 16-fold selectivity toward *T. cruzi* over MRC-5 cells.

The final set of substituents on the piperidine linker were aromatic rings (Table 3, **79**–**82**). This sub-series showed moderate activities with the 3-cyanophenyl (**79**), 4-methylbenzoate (**81**) and methyl-5-picolinate (**82**) all having low micromolar activities (pIC_50_ = 5.7). The 3-pyridinyl analogue (**80**) was less active with a pIC_50_ of 5.2. Best selectivity index was observed for 3-cyanophenyl **79** and methyl-5-picolinate **82**, which both had over 32-fold selectivity.

Overall, the introduction of polar moieties on the phenyl-dihydropyrazolones generally led to low activities against *T. cruzi*. Especially the introduction of a polar moiety directly next to the pyrazolone nitogen such as all carbonyl, sulphonyl and β-alcohol linked moieties (**6**–**10**, **19**, **20**) resulted in analogues with low potencies. Introduction of aliphatic substituents or aromatic moieties resulted in interesting new anti-*T. cruzi* inhibitors with the best compounds **54** and **76** reaching sub micromolar potency. Installing a piperidine linker allowed for the introduction of a variety of substituents and it became apparent that aromatic moieties, either directly, with a methylene bridge or with a 2-atom linker performed best, showing pIC_50_’s around 6.0.

## Conclusion

Our study results in valuable SAR data that has been obtained by introducing a variety of substituents on the dihydropyrazolone nitrogen atom. While decorating this position is synthetically very efficient and a wide variety of chemical functionalities are allowed, our studies did not lead to compounds that have a better activity than NPD-0227. Especially the piperidine linker opens a whole new range of options to introduce substituents and analogues reached low micromolar inhibitory activities against *T. cruzi*. The most active compounds were obtained if the piperidine is substituted with apolar moieties such as aryl or benzyl rings. Introduction of more polar substituents, such as imidazole **25** (cLogP = 2.3), succinimide **46** (cLogP = 0.9) and morpholine **73** (cLogP = 1.6) generally lead to compounds with high micromolar activities. The structural moiety performing best is a 2-atom linker followed by an aromatic moiety, of which sulphonamide **54** (cLogP = 3.8), urea **59** (cLogP = 4.0) and β-alcohols **65** and **66** (cLogP = 3.8/3.9) performed best. These molecules all have activities around 6.0 (pIC_50_) and especially sulphonamide **54** is promising with a pIC50 of 6.2 and >100-fold selectivity over MRC-5 cells. Also other aromatic substituents performed well, such as 4-fluorobenzyl (**76**), 3-cyanophenyl (**79**) and 5-methylnicotinate (**82**) show promising activities around 6.0, with **79** and **82** showing best selectivies (>32-fold) toward *T. cruzi*. While these results of this study are important in the design of novel ‘drug leads’ for Chagas disease, the original goal, of identifying high potency molecules with a lower cLogP did not succeed. Most potent compounds with a cLogP below 2.0 were oxadiazole **33** and sulfamide **51**, which both showed a pIC_50_ of 5.1.

### Experimental Section Biology

*Trypanosoma cruzi in vitro assay.* Bloodstream trypomastigotes of the Y strain of *T. cruzi* were obtained by cardiac puncture of infected Swiss Webster mice on the parasitaemia peak (Meirelles et al., 1986; Batista et al., 2010). For the standard *in vitro* susceptibility assay on intracellular amastigotes, *T. cruzi* Tulahuen CL2, β-galactosidase strain (nifurtimox-sensitive) was used. The strain is maintained on MRC-5_SV2_ (human lung fibroblast) cells in MEM medium, supplemented with 200 mM L-glutamine, 16.5 mM NaHCO_3_ and 5% inactivated fetal calf serum (FCSi). All cultures and assays were conducted at 37°C under an atmosphere of 5% CO_2_. Benznidazole was included in the assays as a positive control.

*MRC-5 cytotoxicity in vitro assay.* MRC-5-_SV2_ cells, originally from a human diploid lung cell line, were cultivated in MEM supplemented with L-glutamine (20 mM), 16.5 mM sodium hydrogen carbonate and 5% FCSi. For the assay, 10^4^ cells/well were seeded onto the test plates containing the pre-diluted sample and incubated at 37°C and 5% CO_2_ for 72 h. Cell viability was assessed fluorometrically 4 h after addition of resazurin (excitation 550 nm, emission 590 nm). The results are expressed as percentage reduction in cell viability compared to untreated controls. Tamoxifen was included in the assays as a positive control.

The use of laboratory rodents was carried out in strict accordance to all mandatory guidelines (EU directives, including the Revised Directive 2010/63/EU on the Protection of Animals used for Scientific Purposes that came into force on January 1, 2013, and the Declaration of Helsinki in its latest version).

### Experimental Section Chemistry

Chemicals and reagents were obtained from commercial suppliers and were used without further purification. Anhydrous DMF, THF, and DCM were obtained by passing them through an activated alumina column prior to use. Microwave reactions were executed using a Biotage^®^ Initiator microwave system. ^1^H NMR spectra were recorded on a Bruker Avance 250 (250 MHz), Bruker Avance 400 (400 MHz), Bruker Avance 500 (500 MHz) or Bruker 600 Avance (600 MHz) spectrometer. Data are reported as follows: chemical shift, integration, multiplicity (s = singlet, d = doublet, dd = double doublet, t = triplet, dt = double triplet, q = quartet, p = pentet, h = heptet, bs = broad singlet, m = multiplet), and coupling constants (Hz). Chemical shifts are reported in ppm with the natural abundance of deuterium in the solvent as the internal reference [CDCl_3_: δ 7.26, (CD_3_)_2_SO: δ 2.50]. ^13^C NMR spectra were recorded on a Bruker Avance 500 (126 MHz) or Bruker Avance 600 (150 MHz). Chemical shifts are reported in ppm with the solvent resonance resulting from incomplete deuteration as the internal reference [CDCl_3_: δ 77.16 or (CD_3_)_2_SO: δ 39.52]. Systematic names for molecules according to IUPAC rules were generated using the Chemdraw AutoName program. LC-MS data was gathered using a Shimadzu HPLC/MS workstation with a LC-20AD pump system, SPD-M20A diode array detection, and a LCMS-2010 EV mass spectrometer. The column used is an Xbridge C18 5 μm column (100 mm × 4.6 mm). Solvents used were the following: solvent B = ACN, 0.1% formic Acid; solvent A = water, 0.1% formic acid. The analysis was conducted using a x?ow rate of 1.0 ml/min, start 5% B, linear gradient to 90% B in 4.5 min, then 1.5 min at 90% B, linear gradient to 5% B in 0.5 min and then 1.5 min at 5% B, total run time of 8 min. All reported compounds have purities >95%, measured at 254 nm, unless otherwise mentioned. All HRMS spectra were recorded on a Bruker micrOTOF mass spectrometer using ESI in positive-ion mode. In case of bromine containing molecules, the lowest peak of ^79^Br was reported. Column puri?cations were either carried out automatically using Biotage equipment or manually, using 60–200 mesh silica. TLC analyses were performed with Merck F254 alumina silica plates using UV visualization. All reactions were done under N_2_ atmosphere, unless specifically mentioned. The cLogPs were calculated using CDD vault, CDD vault uses the ionic logP algorithm.

### Experimental Data

Compound **5**, **6**, **15**, **23**, **27**, **29**, **33**, **38**–**42** and **83** are described below, other compounds can be found in the supporting information.

#### 3-(4-Methoxy-3-(pyridin-3-yl)phenyl)-4,4-dimethyl-1H-pyrazol-5(4H)-one (**5**)

Pyrazolone **4** (1.0 g, 3.4 mmol) and pyridin-3-ylboronic acid (0.62 g, 5.1 mmol) were charged to a microwave tube after which DME (12 ml) and 1 M Na_2_CO_3_ (6.7 ml, 6.7 mmol) were added. The mixture was degassed with N_2_ for 5 m after which Pd (dppf)Cl_2_ (0.28 g, 34 mmol) was added. The reaction was heated at 120°C for 1 h in the microwave. The reaction mixture was diluted with EtOAc (30 ml) and filtered over Celite. The residue was washed with saturated NaHCO_3_ (2 ml × 30 ml) and brine (1 ml × 30 ml). The organic phase was dried over Na_2_SO_4_, filtered and concentrated *in vacuo.* The remaining crude was purified over SiO_2_ using a gradient of 50% EtOAc in heptane toward 5% MeOH in EtOAc to yield 700 mg (2.4 mmol, 70%) of the title compound as a white solid. ^1^H NMR (500 MHz, CDCl_3_) δ 11.50 (s, 1H), 8.69 (s, 1H), 8.55 (d, *J* = 3.8 Hz, 1H), 7.91 (apparent dt, *J* = 7.9, 1.9 Hz, 1H), 7.84 (dd, *J* = 8.7, 2.3 Hz, 1H), 7.73 (d, *J* = 2.3 Hz, 1H), 7.46 (dd, *J* = 7.9, 4.8 Hz, 1H), 7.22 (d, *J* = 8.8 Hz, 1H), 3.83 (s, 3H), 1.37 (s, 6H). ^13^C NMR (126 MHz, CDCl_3_) δ 181.1, 161.6, 157.7, 150.0, 148.6, 137.1, 133.7, 128.1, 128.0, 127.4, 124.4, 123.8, 112.4, 56.3, 46.9, 22.5. LC-MS (ESI) *m*/*z* found: 296 (M + H)^+^; retention time: 2.67 min. HRMS-ESI (M + H)^+^ calculated for C_17_H_18_N_3_O_2_: 296.1394, found: 296.1391.

#### 1-(cyclopropanecarbonyl)-3-(4-Methoxy-3-(Pyridin-3-Yl)phenyl)-4,4-Dimethyl-1h-Pyrazol-5(4H)-One (**6**)

Pyrazolone **5** (50 mg, 0.17 mmol) was dissolved in DMF (2 ml) and sodium hydride (60% in mineral oil) (10 mg, 0.25 mmol) was added. After stirring for 30 min cyclopropanecarbonyl chloride (20 mg, 0.19 mmol) was added and the mixture was stirred at rt for 18 h after which the reaction was quenched with water (25 ml) and extracted with EtOAc (25 ml). The organic layer was washed with sat. aq. NaHCO_3_ (2 ml × 20 ml), brine (20 ml) and dried over MgSO_4_ after which volatiles were evaporated yielding 24 mg (0.07 mmol, 39%) of the title compound as a white solid. ^1^H NMR (500 MHz, CDCl_3_) δ 8.76 (d, *J* = 2.0 Hz, 1H), 8.60 (dd, *J* = 4.8, 1.7 Hz, 1H), 7.95–7.84 (m, 3H), 7.38 (dd, *J* = 7.8, 4.8 Hz, 1H), 7.09–7.03 (m, 1H), 3.89 (s, 3H), 3.05–2.97 (m, 1H), 1.61 (s, 6H), 1.32–1.25 (m, 2H), 1.11–1.02 (m, 2H). ^13^C NMR (126 MHz, CDCl_3_) δ 178.0, 171.9, 162.3, 158.7, 150.0, 148.3, 137.1, 133.3, 129.5, 128.7, 127.8, 123.1, 122.9, 111.2, 55.8, 49.9, 23.2, 13.5, 11.3. LC-MS (ESI) *m*/*z* found: 364 (M + H)^+^; retention time: 3.43 min. HRMS-ESI (M + H)^+^ calculated for C_21_H_22_N_3_O_3_: 364.1656, found: 364.1653.

#### Ethyl 2-(3-(3-bromo-4-methoxyphenyl)-4,4-dimethyl-5-oxo-4,5-dihydro-1H-pyrazol-1-yl)acetate (**13**)

Pyrazolone **4** (5.0 g, 16.8 mmol) was dissolved in DMF (35 ml) and sodium hydride (60% in mineral oil) (0.49 g, 20.2 mmol) was added. After stirring for 30 min ethyl 2-bromoacetate (2.23 ml, 20.2 mmol) was added and the mixture was heated to 50°C for 3 h after which the reaction was quenched with water (250 ml). Solids were filtered off and dried *in vacuo*, yielding 5.6 g (14.6 mmol, 87%) of the title compound as a white solid. ^1^H NMR (500 MHz, CDCl_3_) δ 8.05 (d, *J* = 2.2 Hz, 1H), 7.70 (dd, *J* = 8.6, 2.2 Hz, 1H), 6.93 (d, *J* = 8.7 Hz, 1H), 4.55 (s, 2H), 4.23 (q, *J* = 7.1 Hz, 2H), 3.95 (s, 3H), 1.53 (s, 6H), 1.29 (t, *J* = 7.1 Hz, 3H). ^13^C NMR (126 MHz, CDCl_3_) δ 179.1, 167.8, 160.9, 157.2, 131.4, 126.7, 124.6, 112.3, 111.5, 61.7, 56.4, 48.1, 45.8, 22.5, 14.1. LC-MS (ESI) *m*/*z* found: 383 (M + H)^+^; retention time: 4.75 min. HRMS-ESI (M + H)^+^ calculated for C_16_H_20_BrN_2_O_4_: 383.0601, found: 383.0588.

#### 3-(3-Bromo-4-methoxyphenyl)-1-(cyclohexylmethyl)-4,4-dimethyl-1H-pyrazol-5(4H)-one (**15**)

Pyrazolone **4** (100 mg, 0.30 mmol) was dissolved in DMF (3 ml) and sodium hydride (60% in mineral oil) (15 mg, 0.38 mmol) was added. After stirring for 30 min (bromomethyl)cyclohexane (63 mg, 0.67 mmol) was added and the mixture was stirred at rt for 18 h after which the reaction was quenched with water (25 ml) and extracted with EtOAc (25 ml). The organic layer was washed with brine (2 ml × 20 ml) and dried over MgSO_4_ after which volatiles were evaporated yielding 105 mg (0.27 mmol, 91%) of the title compound as a transparent oil. ^1^H NMR (500 MHz, CDCl_3_) δ 8.03 (d, *J* = 2.2 Hz, 1H), 7.66 (dd, *J* = 8.6, 2.2 Hz, 1H), 6.90 (d, *J* = 8.7 Hz, 1H), 3.92 (s, 3H), 3.56 (d, *J* = 7.2 Hz, 2H), 1.89–1.80 (m, 1H), 1.74–1.68 (m, 2H), 1.67–1.61 (m, 3H), 1.45 (s, 6H), 1.28–1.13 (m, 5H), 1.08–0.89 (m, 2H). ^13^C NMR (126 MHz, CDCl_3_) δ 178.6, 160.1, 156.9, 131.2, 126.5, 125.0, 112.3, 111.5, 56.4, 50.3, 48.3, 36.9, 30.5, 26.4, 25.7, 22.8. LC-MS (ESI) *m*/*z* found: 393 (M + H)^+^; retention time: 6.05 min. HRMS-ESI (M + H)^+^ calculated for C_19_H_26_BrN_2_O_2_: 393.1172, found: 393.1156.

#### 1-(cyclohexylmethyl)-3-(4-Methoxy-3-(Pyridin-3-Yl)phenyl)-4,4-Dimethyl-1h-Pyrazol-5(4H)-One (**23**)

Pyrazolone **15** (100 mg, 0.16 mmol) and pyridin-3-ylboronic acid (47 mg, 0.38 mmol) were charged to a microwave tube after which DME (3 ml) and 1 M Na_2_CO_3_ (0.8 ml, 0.8 mmol) were added. The mixture was degassed with N_2_ for 5 m after which Pd (dppf) Cl_2_ (21 mg, 25 µmol) was added. The reaction was heated at 120°C for 1 h in the microwave. The reaction mixture was diluted with EtOAc (30 ml) and filtered over Celite. The residue was washed with saturated NaHCO_3_ (2 ml × 30 ml) and brine (1 ml × 30 ml). The organic phase was dried over Na_2_SO_4_, filtered and concentrated *in vacuo.* The remaining crude was purified over SiO_2_ using a gradient of 50% EtOAc in heptane toward 100% EtOAc to yield 64 mg (0.16 mmol, 64%) of the title compound as a white solid. ^1^H NMR (500 MHz, CDCl_3_) δ 8.78 (d, *J* = 2.1 Hz, 1H), 8.59 (d, *J* = 4.8 Hz, 1H), 7.88 (apparent dt, *J* = 7.8, 1.9 Hz, 1H), 7.82 (d, *J* = 2.2 Hz, 1H), 7.77 (dd, *J* = 8.6, 2.2 Hz, 1H), 7.37 (dd, *J* = 7.9, 4.9 Hz, 1H), 7.03 (d, *J* = 8.6 Hz, 1H), 3.87 (s, 3H), 3.58 (d, *J* = 7.2 Hz, 2H), 2.05–1.79 (m, 2H), 1.76–1.60 (m, 5H), 1.50 (s, 6H), 1.29–1.10 (m, 3H), 1.06–0.95 (m, 2H). ^13^C NMR (126 MHz, CDCl_3_) δ 178.7, 161.0, 157.8, 150.1, 148.2, 137.0, 133.6, 128.7, 127.6, 127.5, 124.2, 123.1, 111.1, 55.8, 50.3, 48.4, 36.9, 30.5, 26.4, 25.7, 22.9. LC-MS (ESI) *m*/*z* found: 392 (M + H)^+^; retention time: 4.54 min. HRMS-ESI (M + H)^+^ calculated for C_24_H_30_N_3_O_2_: 392.2333, found: 392.2319.

#### 2-(3-(3-bromo-4-methoxyphenyl)-4,4-dimethyl-5-oxo-4,5-dihydro-1H-pyrazol-1-yl)acetohydrazide (**27**)

Ester **13** (1.5 g, 3.91 mmol) was refluxed in hydrazine monohydrate (1.89 ml, 39.1 mmol) and ethanol (25 ml) for 18 h. After that the mixture was allowed to cool down, the mixture was concentrated and 20 ml of water was added. The solids were filtered off and dried *in vacuo* yielding 1.36 g (3.7 mmol, 94%) of the title compound as a white solid. ^1^H NMR (500 MHz, DMSO-*d*6) δ 9.29 (s, 1H), 7.96 (s, 1H), 7.79 (d, *J* = 8.6 Hz, 1H), 7.17 (d, *J* = 8.6 Hz, 1H), 4.29 (d, *J* = 13.2 Hz, 4H), 3.90 (s, 3H), 1.39 (s, 6H). ^13^C NMR (126 MHz, DMSO-d_6_) δ 178.9, 166.4, 160.0, 157.0, 130.5, 127.6, 124.6, 113.1, 111.8, 56.9, 47.7, 45.9, 22.4. LC-MS (ESI) *m*/*z* found:369 (M + H)^+^; retention time: 3.59 min.

#### 1-((1,3,4-Oxadiazol-2-yl)methyl)-3-(3-bromo-4-methoxyphenyl)-4,4-dimethyl-1H-pyrazol-5(4H)-one (**29**)

Hydrazide **27** (200 mg, 0.54 mmol) was stirred in triethylorthoformate (0.9 ml, 5.4 mmol) and PTSA (10.30 mg, 0.054 mmol) was added. The mixture was heated at 80°C for 18 h after which the volatiles were evaporated and the resulting crude was purified over SiO_2_ using a gradient of 20% EtOAc in heptane toward 80% EtOAc in heptane. This yielded 70 mg (0.19 mmol, 34%) of the title compound as a white solid. ^1^H NMR (500 MHz, CDCl_3_) δ 8.42 (s, 1H), 7.99 (d, *J* = 2.2 Hz, 1H), 7.66 (dd, *J* = 8.7, 2.2 Hz, 1H), 6.90 (d, *J* = 8.7 Hz, 1H), 5.22 (s, 2H), 3.92 (s, 3H), 1.52 (s, 6H). ^13^C NMR (126 MHz, CDCl_3_) δ 178.5, 161.9, 161.6, 157.4, 153.6, 131.4, 126.9, 124.2, 112.3, 111.6, 56.4, 48.1, 39.0, 22.6. LC-MS (ESI) *m*/*z* found: 397 (M + H)^+^; retention time: 4.27 min. HRMS-ESI (M + H)^+^ calculated for C_15_H_16_BrN_4_O_3_: 379.0400, found: 379.0391.

#### 1-((1,3,4-Oxadiazol-2-yl)methyl)-3-(4-methoxy-3-(pyridin-3-yl)phenyl)-4,4-dimethyl-1H-pyrazol-5(4H)-one (**33**)

Pyrazolone **29** (50 mg, 0.13 mmol) and pyridin-3-ylboronic acid (24 mg, 0.38 mmol) were charged to a microwave tube after which DME (3 ml) and 1 M Na_2_CO_3_ (0.5 ml, 0.5 mmol) were added. The mixture was degassed with N_2_ for 5 m after which Pd (dppf) Cl_2_ (11 mg, 13 µmol) was added. The reaction was heated at 120°C for 1 h in the microwave. The reaction mixture was diluted with EtOAc (30 ml) and filtered over Celite. The residue was washed with saturated NaHCO_3_ (2 ml × 30 ml) and brine (1 ml × 30 ml). The organic phase was dried over Na_2_SO_4_, filtered and concentrated *in vacuo.* The remaining crude was purified over SiO_2_ using a gradient of 50% EtOAc in heptane toward 100% EtOAc to yield 36 mg (0.10 mmol, 72%) of the title compound as a white solid. ^1^H NMR (500 MHz, CDCl_3_) δ 8.79 (s, 1H), 8.61 (s, 1H), 8.41 (s, 1H), 7.95–7.83 (m, 1H), 7.83–7.73 (m, 2H), 7.41 (s, 1H), 7.03 (d, *J* = 8.4 Hz, 1H), 5.23 (s, 2H), 3.87 (s, 3H), 1.56 (s, 6H). ^13^C NMR (126 MHz, CDCl_3_) δ 178.6, 162.4, 162.0, 158.2, 153.6, 149.5, 147.6, 137.5, 133.9, 128.9, 128.0, 127.4, 123.4, 111.2, 55.9, 48.2, 39.0, 22.8. LC-MS (ESI) *m*/*z* found: 378 (M + H)^+^; retention time: 2.93 min. HRMS-ESI (M + H)^+^ calculated for C_20_H_20_N_5_O_3_: 378.1562, found: 378.1562.

#### 3-(3-Bromo-4-methoxyphenyl)-4,4-dimethyl-1-(piperidin-4-yl)-1H-pyrazol-5(4H)-one (**38**)

Keto-ester **37** (32 g, 102 mmol) was dissolved in methanol (200 ml) and stirred. To this solution 4-hydrazinylpiperidine dihydrochloride (76 g, 406 mmol) dissolved in Water (80 ml) was added. The mixture was refluxed for 72 h after which 150 ml water was added and MeOH was removed *in vacuo*. To the remaining solution 10 M NaOH was added slowly until the pH was ∼13. Solids were collected and washed with water (20 ml) to yield 31 g (82 mmol, 80%) of the title compound. ^1^H NMR (500 MHz, CDCl_3_) δ 8.00 (d, *J* = 2.2 Hz, 1H), 7.78 (dd, *J* = 8.6, 2.2 Hz, 1H), 7.16 (d, *J* = 8.8 Hz, 1H), 4.06–3.94 (m, 1H), 3.90 (s, 3H), 3.04–2.93 (m, 2H), 2.53–2.51 (m, 2H), 1.84–1.75 (m, 2H), 1.66–1.56 (m, 2H), 1.35 (s, 6H). ^13^C NMR (126 MHz, CDCl_3_) δ 177.5, 159.7, 156.9, 130.4, 127.4, 124.8, 113.1, 111.9, 56.9, 51.5, 48.4, 45.6, 31.7, 22.4. LC-MS (ESI) *m*/*z* found: 380 (M + H)^+^; retention time: 3.19 min. HRMS-ESI (M + H)^+^ calculated for C_17_H_23_BrN_3_O_2_: 380.0968, found: 380.0968.

#### Tert-butyl 4-(3-(3-bromo-4-methoxyphenyl)-4,4-dimethyl-5-oxo-4,5-dihydro-1H-pyrazol-1-yl)piperidine-1-carboxylate (**39**)

Piperidine **38** (25 g, 65.7 mmol) was added to a round-bottom flask after which DCM (500 ml) was added, followed by di-tert-butyl dicarbonate (15.6 ml, 67.1 mmol) and triethylamine (9.4 ml, 67.1 mmol). The reaction mixture was extracted with water (2 ml × 400 ml) and brine (400 ml) and the organic layer was dried over Na_2_SO_4_. Solids were filtered off and volatiles were evaporated yielding 28 g (58 mmol, 89%) of the title compound as a light-brown solid. ^1^H NMR (500 MHz, CDCl_3_) δ 8.05 (d, *J* = 2.1 Hz, 1H), 7.70 (dd, *J* = 8.7, 2.2 Hz, 1H), 6.94 (d, *J* = 8.6 Hz, 1H), 4.31–4.20 (m, 3H), 3.96 (s, 3H), 2.87 (t, *J* = 12.9 Hz, 2H), 2.03 (apparent qd, *J* = 12.5, 4.5 Hz, 2H), 1.83–1.78 (m, 2H), 1.51 (s, 9H), 1.48 (s, 6H). LC-MS (ESI) *m*/*z* found: 424 (M + H, -t-Bu)^+^; retention time: 5.34 min. HRMS-ESI (M + H)^+^ calculated for C_22_H_31_N_3_O_4_: 480.1492, found: 480.1477.

#### Tert-butyl 4-(3-(4-methoxy-3-(pyridin-3-yl)phenyl)-4,4-dimethyl-5-oxo-4,5-dihydro-1H-pyrazol-1-yl)piperidine-1-carboxylate (**40**)

Boc-protected piperidine **39** (25.2 g, 52.5 mmol) and pyridin-3-ylboronic acid (9.0 g, 73 mmol) were charged to a round-bottom flask after which DME (400 ml) and 1 M Na_2_CO_3_ (210 ml, 210 mmol) were added. The mixture was degassed with N_2_ for 15 m after which Pd (dppf) Cl_2_ (2.1 g, 2.6 mmol) was added. The reaction was heated at 80°C for 16 h. The reaction mixture was diluted with MTBE (500 ml) and filtered over Celite. The residue was washed with saturated NaHCO_3_ (2 ml × 600 ml) and brine (1 ml × 600 ml). The organic phase was dried over Na_2_SO_4_, filtered and concentrated *in vacuo* to yield 21.5 g (44.9 mmol, 86%) of the title compound. ^1^H NMR (500 MHz, CDCl_3_) δ 8.75 (s, 1H), 8.56 (d, *J* = 4.0 Hz, 1H), 7.84 (d, *J* = 7.9 Hz, 1H), 7.78 (d, *J* = 2.0 Hz, 1H), 7.75 (dd, *J* = 8.6, 2.1 Hz, 1H), 7.35 (dd, *J* = 7.7, 4.9 Hz, 1H), 7.01 (d, *J* = 8.7 Hz, 1H), 4.33–4.08 (m, 3H), 3.85 (s, 3H), 2.82 (s, 2H), 1.99 (apparent q, *J* = 11.2 Hz, 2H), 1.76 (d, *J* = 11.6 Hz, 2H), 1.47 (s, 7H), 1.45 (s, 9H). ^13^C NMR (126 MHz, CDCl_3_) δ 178.0, 161.2, 157.8, 154.7, 150.1, 148.3, 136.8, 133.5, 128.7, 127.7, 127.5, 124.0, 123.1, 111.1, 79.7, 55.8, 50.8, 48.8, 30.0, 28.4, 22.7. LC-MS (ESI) *m*/*z* found: 479 (M + H)^+^; retention time: 4.03 min. HRMS-ESI (M + H)^+^ calculated for C_27_H_35_N_4_O_4_: 479.2653, found: 479.2651.

#### 3-(4-Methoxy-3-(pyridin-3-yl)phenyl)-4,4-dimethyl-1-(piperidin-4-yl)-1H-pyrazol-5(4H)-one Hydrochloride (**41**)

Boc-protected piperidine **40** (25.1 g, 52.4 mmol) was dissolved in dioxane (100 ml) and hydrogen chloride in dioxane (4 N) (131 ml, 524 mmol) was added in portions. The reaction was stirred for 40 h after which volatiles were evaporated. Attempts were made to recrystallize the resulting dark-oil from MTBE, iPr-OH, iPr-OH:H_2_O to no avail. The crude was redissolved in EtOAc (800 ml) and extracted with aq. sat. Na_2_CO_3_ (2 ml × 500 ml) and brine (500 ml). The organic layer was dried over Na_2_SO_4_ and volatiles were evaporated. Attempts to recrystallize from i-PrOH or iPrOH:H2O were again unsuccessful. Evaporation of i-PrOH:H_2_O ultimately yielded 16.7 g (44.1 mmol, 84%) of the title compound as a light brown powder. ^1^H NMR (500 MHz, CDCl_3_) δ 8.70–8.67 (m, 1H), 8.56 (dd, *J* = 4.9, 1.6 Hz, 1H), 7.91 (apparent dt, *J* = 8.0, 1.9 Hz, 1H), 7.85 (dd, *J* = 8.7, 1.9 Hz, 1H), 7.76 (d, *J* = 1.8 Hz, 1H), 7.47 (dd, *J* = 7.7, 4.9 Hz, 1H), 7.23 (d, *J* = 8.8 Hz, 1H), 4.08–3.96 (m, 1H), 3.84 (s, 3H), 3.00 (d, *J* = 12.3 Hz, 2H), 2.58–2.51 (m, 2H), 1.81 (apparent qd, *J* = 12.3, 4.1 Hz, 2H), 1.68–1.58 (m, 2H), 1.39 (s, 6H). ^13^C NMR (126 MHz, CDCl_3_) δ 177.2, 160.3, 157.5, 149.7, 149.6, 148.3, 148.2, 136.7, 133.2, 127.8, 127.2, 123.5, 123.4, 112.0, 55.9, 51.0, 48.0, 45.2, 31.2, 22.2. LC-MS (ESI) *m*/*z* found: 379 (M + H)^+^; retention time: 2.40 min. HRMS-ESI (M + H)^+^ calculated for C_22_H_27_N_4_O_2_: 379.2129, found: 379.2121.

#### 1-(1-(cyclopropanecarbonyl)piperidin-4-yl)-3-(4-methoxy-3-(pyridin-3-yl)phenyl)-4,4-dimethyl-1H-pyrazol-5(4H)-one (**42**)

Amine **41** (100 mg, 0.26 mmol) was stirred with sodium hydride (60% in mineral oil) (16 mg, 0.40 mmol) in DMF (1 ml). After 30 min cyclopropanecarbonyl chloride (0.026 ml, 0.29 mmol) was added. The reaction was stirred for 2 h after which the reaction was quenched with water (20 ml) and extracted with EtOAc (20 ml). The organic layer was washed with sat. aq. NaHCO_3_ solution (2 ml × 20 ml), brine (20 ml) and dried over MgSO_4_. Volatiles were evaporated and the remaining crude was purified over SiO_2_ using a gradient of 50% EtOAc in heptane toward 5% MeOH in EtOAc, yielding 45 mg (0.10 mmol, 38%) of the title compound as a white solid. ^1^H NMR (500 MHz, CDCl_3_) δ 8.78 (s, 1H), 8.60 (d, *J* = 4.0 Hz, 1H), 7.89 (d, *J* = 7.9 Hz, 1H), 7.80 (d, *J* = 2.2 Hz, 1H), 7.77 (dd, *J* = 8.6, 2.2 Hz, 1H), 7.41 (dd, *J* = 7.7, 4.9 Hz, 1H), 7.03 (d, *J* = 8.7 Hz, 1H), 4.75 (d, *J* = 12.9 Hz, 1H), 4.41–4.29 (m, 2H), 3.88 (s, 3H), 3.24 (t, *J* = 12.7 Hz, 1H), 2.72 (apparent t, *J* = 12.4 Hz, 1H), 2.14–1.74 (m, 7H), 1.50 (s, 6H), 1.05–0.95 (m, 2H), 0.77 (dd, *J* = 7.9, 3.5 Hz, 2H). ^13^C NMR (126 MHz, CDCl_3_) δ 178.0, 171.9, 161.3, 157.8, 149.8, 148.0, 137.2, 133.7, 128.7, 127.6, 127.5, 124.0, 123.3, 111.2, 55.8, 50.7, 48.8, 44.7, 41.4, 30.8, 29.7, 22.8, 11.1, 7.5, 7.4. LC-MS (ESI) *m*/*z* found: 447 (M + H)^+^; retention time: 3.20 min. HRMS-ESI (M + H)^+^ calculated for C_26_H_31_N_4_O_3_: 447.2391, found: 447.2390.

#### 3-(4-Methoxy-3-(pyridin-3-yl)phenyl)-4,4-dimethyl-1-(1-(morpholine-4-carbonyl)piperidin-4-yl)-1H-pyrazol-5(4H)-one (**83**)

Amine **41** (400 mg, 1.1 mmol) was dissolved in dioxane (15 ml) and 25 ml of saturated NaHCO_3_ was added followed by a dropwise addition of a 4-nitrophenyl chloroformate (639 mg, 3.2 mmol) in dioxane (10 ml) solution. The reaction mixture was stirred for 22 h, diluted with EtOAc and washed with a saturated sodium bicarbonate solution. The organic fraction was dried with brine, evaporated to dryness in the presence of SiO_2_ and purified over the same medium using 1:3 EtOAc:c-hexane with 1% TEA toward 100% EtOAc with 1% TEA. After confirming identify of the product by semi-crude NMR it was used in the following step. K_2_CO_3_ (25 mg, 0.18 mmol) and morpholine (0.016 ml, 0.18 mmol) were dissolved in DMF (5 ml) to which the earlier isolated intermediate (50 mg, 0.092 mmol) was added. This reaction mixture was stirred at room temperature for 4 days and an additional equivalent morpholine (0.016 ml, 0.18 mmol) was added after 2 days. The reaction mixture was diluted with EtOAc (25 ml) and washed with sat. aq. Na_2_CO_3_ (25 ml). The aqueous layer was back extracted with EtOAc (25 ml) and the combined organic layers were washed with brine (25 ml), dried over Na_2_SO_4_ and concentrated *in vacuo*. The remaining crude was coated on SiO_2_ and purified over SiO_2_ using a gradient from EtOAc (1% TEA): cyclohexane 1:1 (v/v) toward 1% MeOH in EtOAc (1% TEA), evaporation of volatiles yielded a colourless oil which was re-dissolved in DCM and concentrated to afford 19 mg (0.039 mmol, 43%). ^1^H NMR (600 MHz, CDCl_3_) δ 8.83–8.78 (m, 1H), 8.62–8.59 (m, 1H), 8.04–7.98 (m, 1H), 7.82 (d, *J* = 2.2 Hz, 1H), 7.77 (dd, *J* = 8.7, 2.2 Hz, 1H), 7.49 (dd, *J* = 7.8, 5.1 Hz, 1H), 7.03 (d, *J* = 8.7 Hz, 1H), 4.29–4.23 (m, 1H), 3.87 (s, 3H), 3.85–3.80 (m, 2H), 3.70–3.63 (m, 4H), 3.31–3.25 (m, 4H), 2.94–2.83 (m, 2H), 2.10–2.02 (m, 2H), 1.85–1.78 (m, 2H), 1.49 (s, 6H). ^13^C NMR (151 MHz, CDCl_3_) δ 178.0, 163.9, 161.1, 157.8, 148.5, 146.5, 138.5, 134.4, 128.7, 128.0, 126.9, 124.2, 123.7, 111.2, 66.7, 55.8, 51.0, 48.8, 47.4, 46.1, 30.0, 22.7. LC-MS (ESI) *m/z* found: 492 (M + H)^+^; retention time: 3.07 min. HRMS-ESI (M + H)^+^ calculated for C_27_H_34_N_5_O_4_^+^: 492.2605, found 492.2615.

## Data Availability

The original contributions presented in the study are included in the article/Supplementary Material, further inquiries can be directed to the corresponding author.

## Glossary

ACN: acetonitrile
AcOH: acetic acid
BINAP: 2,2′-bis(diphenylphosphino)-1.,1′-binaphthyl
bs: broad singlet
d: days
d: doublet
DBN: 1,5-Diazabicyclo[4.3.0]non-5-ene
DCE: dichlorethane
DCM: dichloromethane
dd: double doublet
DIPEA: *N*,*N*-diisopropylethylamine
DMAP: 4-dimethylaminopyridine
DME: dimethoxyethane
DMF: dimethylformamide
DMSO: dimethylsulfoxide
dt: double triplet
DTU: discrete typing unit
EDCI: 1-Ethyl-3-(3-dimethylaminopropyl)carbodiimide
ESI: electron spray ionization
EtOAc: ethylacetate
EtOH: ethanol
FCS: fetal calf serum
h: heptet: hour
h: heptet: hour
HOBt: hydroxybenzotriazole
HPLC: high pressure liquid chromatography
HRMS: high resolution mass spectroscopy
Hz: Hertz
i-PrOH: isopropanol
LC-MS: liquid chromatography mass spectroscopy
m: minute
m: multiplet
MeOH: methanol
MRC-5: medical research council strain 5
MTBE: methyl tertbutyl ether
NaOtBu: sodium tert-butoxide
o.n.: overnight
p: pentet
PDE4NPD: phosphodiesterase inhibitors for neglected parasitic disease
pIC_50_: -log of the value at which 50% of growth is inhibited
rt: room temperature
s: singlet
SAR: structure activity relationship
t: triplet
T3P: propylphosphonic anhydride
Tcr: *Trypanosoma cruzi*
TEA: triethylamine
q: quartet
THF: tetrahydrofuran
TLC: thin layer chromatography
UV: ultraviolet
